# 2,2′-Bipyridine-5,5′-dicarboxylic acid

**DOI:** 10.1107/S1600536809030207

**Published:** 2009-08-08

**Authors:** Chongchen Wang

**Affiliations:** a School of Environmental and Energy Engineering, Beijing University of Civil Engineering and Architecture, 100044 Beijing, People’s Republic of China

## Abstract

The title mol­ecule, C_12_H_8_N_2_O_4_, lies on an inversion center. In the crystal structure, inter­molecular O—H⋯O hydrogen bonds connect mol­ecules into one-dimensional chains along [1
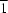
1].

## Related literature

For synthetic applications of the title compound, see: Schokecht & Kempe (2004 ▶). 
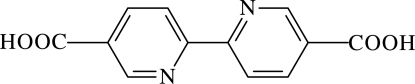


## Experimental

### 

#### Crystal data

C_12_H_8_N_2_O_4_
*M* *_r_* = 244.20
Triclinic, 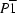

*a* = 3.7384 (5) Å
*b* = 6.3934 (8) Å
*c* = 10.7786 (13) Å
α = 98.774 (2)°
β = 92.567 (1)°
γ = 90.000 (1)°
*V* = 254.34 (6) Å^3^
*Z* = 1
Mo *K*α radiation
μ = 0.12 mm^−1^
*T* = 298 K
0.15 × 0.11 × 0.08 mm

#### Data collection

Bruker SMART CCD diffractometer
Absorption correction: multi-scan (*SADABS*; Sheldrick, 1996 ▶) *T*_min_ = 0.982, *T*_max_ = 0.990
1343 measured reflections
893 independent reflections
657 reflections with *I* > 2σ(*I*)
*R*_int_ = 0.023

#### Refinement

*R*[*F* ^2^ > 2σ(*F* ^2^)] = 0.071
*wR*(*F* ^2^) = 0.214
*S* = 1.14
893 reflections
82 parameters
H-atom parameters constrained
Δρ_max_ = 0.33 e Å^−3^
Δρ_min_ = −0.36 e Å^−3^

### 

Data collection: *SMART* (Bruker, 2007 ▶); cell refinement: *SAINT* (Bruker, 2007 ▶); data reduction: *SAINT*; program(s) used to solve structure: *SHELXS97* (Sheldrick, 2008 ▶); program(s) used to refine structure: *SHELXL97* (Sheldrick, 2008 ▶); molecular graphics: *SHELXTL* (Sheldrick, 2008 ▶) and *PLATON* (Spek, 2009 ▶); software used to prepare material for publication: *SHELXTL*.

## Supplementary Material

Crystal structure: contains datablocks global, I. DOI: 10.1107/S1600536809030207/lh2868sup1.cif

Structure factors: contains datablocks I. DOI: 10.1107/S1600536809030207/lh2868Isup2.hkl

Additional supplementary materials:  crystallographic information; 3D view; checkCIF report

## Figures and Tables

**Table 1:** Hydrogen-bond geometry (Å, °)

*D*—H⋯*A*	*D*—H	H⋯*A*	*D*⋯*A*	*D*—H⋯*A*
O1—H1⋯O2^i^	0.82	1.82	2.625 (3)	168

Symmetry code: (i) 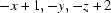.

**Fig. 1.**
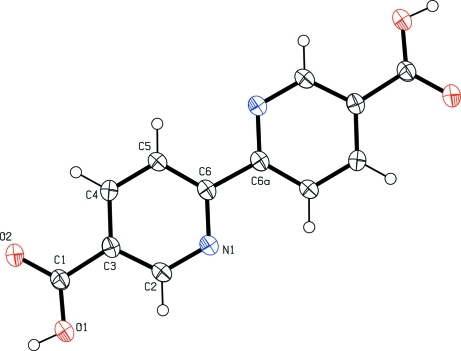
The molecular structure of the title compound shown with 30% probabilty ellipsoids [symmetry code: (a) -x, -y+1, -z+1].

**Fig. 2.**
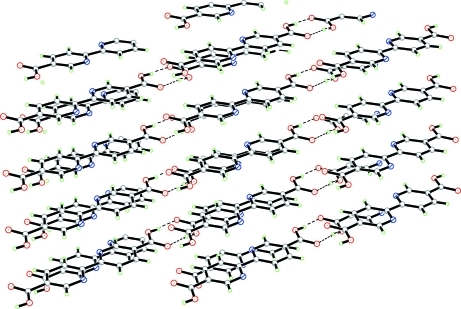
Part of the crystal structure of the title compound with hydrogen bonds shown as dashed lines. The one-dimensional hydrogen-bonded chains propagate along [1-11].

## supplementary crystallographic information

### Comment

2,2'-bipyridine-5,5'-dicarboxylate acid is a potential multi-dentate ligand with a versatile coordination mode, which has been used in self-assembled porous coordination synthesis (Schokecht & Kempe, 2004). The crystals of the title compound were obtained unintentionally as the harvested product of the hydrothermal reaction of 2,2'-bipyridine-5,5'-dicarboxylate acid, Eu_2_O_3_ and 1,10-phenanthroline.

The molecular structure of the title compound is shown in Fig. 1. In the crystal structure, intermolecular O—H···O hydrogen bonds connect molecules into one-dimensional chains along [1 -1 1] (Fig. 2).

### Experimental

Yellow needle-like crystals of the title compound were obtained by hydrothermal reaction of 2,2'-bipyridine-5,5'-dicarboxylate acid (0.04884 g), 1,10-phenanthroline (0.0360 g), Eu_2_O_3_ (0.0702 g) and deionized water (15 ml) in a 23 ml teflon-lined reaction vesset at 433 K for 120 h, followed by slow cooling to room temperature.

### Refinement

All H atoms were placed in calculated positions and included in a riding-model approximation, with C—H = 0.93 Å, O-H = 0.82Å and *U*_iso_(H)= 1.2*U*_eq_(C) or 1.5*U*_eq_(O).

### Crystal data

**Table:**

C_12_H_8_N_2_O_4_	*Z* = 1
*M**_r_* = 244.20	*F*(000) = 126
Triclinic, *P*1	*D*_x_ = 1.594 Mg m^−^^3^
Hall symbol: -P 1	Mo *K*α radiation, λ = 0.71073 Å
*a* = 3.7384 (5) Å	Cell parameters from 528 reflections
*b* = 6.3934 (8) Å	θ = 3.2–27.6°
*c* = 10.7786 (13) Å	µ = 0.12 mm^−^^1^
α = 98.774 (2)°	*T* = 298 K
β = 92.567 (1)°	Needle, yellow
γ = 90.000 (1)°	0.15 × 0.11 × 0.08 mm
*V* = 254.34 (6) Å^3^	

### Data collection

**Table:**

Bruker SMART CCD diffractometer	893 independent reflections
Radiation source: fine-focus sealed tube	657 reflections with *I* > 2σ(*I*)
graphite	*R*_int_ = 0.023
φ and ω scans	θ_max_ = 25.0°, θ_min_ = 1.9°
Absorption correction: multi-scan (*SADABS*; Sheldrick, 1996)	*h* = −4→4
*T*_min_ = 0.982, *T*_max_ = 0.990	*k* = −7→7
1343 measured reflections	*l* = −11→12

### Refinement

**Table:**

Refinement on *F*^2^	Primary atom site location: structure-invariant direct methods
Least-squares matrix: full	Secondary atom site location: difference Fourier map
*R*[*F*^2^ > 2σ(*F*^2^)] = 0.071	Hydrogen site location: inferred from neighbouring sites
*wR*(*F*^2^) = 0.214	H-atom parameters constrained
*S* = 1.14	*w* = 1/[σ^2^(*F*_o_^2^) + (0.1116*P*)^2^ + 0.1159*P*] where *P* = (*F*_o_^2^ + 2*F*_c_^2^)/3
893 reflections	(Δ/σ)_max_ < 0.001
82 parameters	Δρ_max_ = 0.33 e Å^−^^3^
0 restraints	Δρ_min_ = −0.36 e Å^−^^3^

### Special details

**Table:**

Geometry. All e.s.d.'s (except the e.s.d. in the dihedral angle between two l.s. planes) are estimated using the full covariance matrix. The cell e.s.d.'s are taken into account individually in the estimation of e.s.d.'s in distances, angles and torsion angles; correlations between e.s.d.'s in cell parameters are only used when they are defined by crystal symmetry. An approximate (isotropic) treatment of cell e.s.d.'s is used for estimating e.s.d.'s involving l.s. planes.
Refinement. Refinement of *F*^2^ against ALL reflections. The weighted *R*-factor *wR* and goodness of fit *S* are based on *F*^2^, conventional *R*-factors *R* are based on *F*, with *F* set to zero for negative *F*^2^. The threshold expression of *F*^2^ > σ(*F*^2^) is used only for calculating *R*-factors(gt) *etc*. and is not relevant to the choice of reflections for refinement. *R*-factors based on *F*^2^ are statistically about twice as large as those based on *F*, and *R*- factors based on ALL data will be even larger.

### Fractional atomic coordinates and isotropic or equivalent isotropic displacement parameters (Å^2^)

**Table:**

	*x*	*y*	*z*	*U*_iso_*/*U*_eq_	
N1	0.1979 (7)	0.2556 (4)	0.5341 (2)	0.0351 (8)	
O1	0.5129 (7)	−0.0316 (4)	0.8383 (2)	0.0492 (8)	
H1	0.5614	−0.0788	0.9036	0.074*	
O2	0.2843 (7)	0.2274 (4)	0.9736 (2)	0.0536 (9)	
C1	0.3541 (8)	0.1439 (5)	0.8636 (3)	0.0334 (8)	
C2	0.2905 (8)	0.1631 (5)	0.6337 (3)	0.0347 (9)	
H2	0.3922	0.0293	0.6197	0.042*	
C3	0.2438 (7)	0.2552 (5)	0.7571 (3)	0.0307 (9)	
C4	0.0964 (8)	0.4566 (5)	0.7784 (3)	0.0353 (9)	
H4	0.0640	0.5238	0.8598	0.042*	
C5	−0.0008 (8)	0.5548 (5)	0.6768 (3)	0.0332 (8)	
H5	−0.1009	0.6892	0.6890	0.040*	
C6	0.0520 (8)	0.4512 (5)	0.5562 (3)	0.0295 (8)	

### Atomic displacement parameters (Å^2^)

**Table:**

	*U*^11^	*U*^22^	*U*^33^	*U*^12^	*U*^13^	*U*^23^
N1	0.0443 (16)	0.0333 (15)	0.0284 (15)	0.0064 (12)	−0.0011 (12)	0.0079 (12)
O1	0.0724 (18)	0.0463 (16)	0.0308 (14)	0.0208 (13)	−0.0006 (12)	0.0129 (11)
O2	0.083 (2)	0.0525 (17)	0.0267 (14)	0.0231 (14)	0.0056 (12)	0.0108 (11)
C1	0.0347 (17)	0.0366 (18)	0.0297 (17)	0.0043 (14)	−0.0014 (13)	0.0088 (14)
C2	0.0390 (18)	0.0339 (18)	0.0329 (18)	0.0076 (14)	−0.0009 (13)	0.0108 (14)
C3	0.0309 (16)	0.0350 (19)	0.0271 (18)	0.0003 (14)	−0.0020 (13)	0.0083 (14)
C4	0.0446 (19)	0.0375 (19)	0.0240 (16)	0.0068 (15)	0.0024 (13)	0.0047 (13)
C5	0.0393 (18)	0.0321 (17)	0.0296 (17)	0.0078 (14)	0.0009 (13)	0.0092 (14)
C6	0.0288 (15)	0.0318 (18)	0.0289 (17)	−0.0009 (13)	−0.0021 (12)	0.0088 (14)

### Geometric parameters (Å, °)

**Table:**

N1—C2	1.335 (4)	C2—H2	0.9300
N1—C6	1.357 (4)	C3—C4	1.391 (4)
O1—C1	1.267 (4)	C4—C5	1.378 (4)
O1—H1	0.8200	C4—H4	0.9300
O2—C1	1.263 (4)	C5—C6	1.388 (4)
C1—C3	1.484 (4)	C5—H5	0.9300
C2—C3	1.388 (4)	C6—C6^i^	1.482 (6)
			
C2—N1—C6	117.4 (3)	C4—C3—C1	120.8 (3)
C1—O1—H1	109.5	C5—C4—C3	118.9 (3)
O2—C1—O1	123.7 (3)	C5—C4—H4	120.5
O2—C1—C3	118.7 (3)	C3—C4—H4	120.5
O1—C1—C3	117.6 (3)	C4—C5—C6	119.3 (3)
N1—C2—C3	123.8 (3)	C4—C5—H5	120.3
N1—C2—H2	118.1	C6—C5—H5	120.3
C3—C2—H2	118.1	N1—C6—C5	122.4 (3)
C2—C3—C4	118.2 (3)	N1—C6—C6^i^	116.1 (3)
C2—C3—C1	121.0 (3)	C5—C6—C6^i^	121.5 (4)
			
C6—N1—C2—C3	0.4 (5)	C2—C3—C4—C5	0.8 (5)
N1—C2—C3—C4	−0.9 (5)	C1—C3—C4—C5	179.7 (3)
N1—C2—C3—C1	−179.8 (3)	C3—C4—C5—C6	−0.3 (5)
O2—C1—C3—C2	−175.3 (3)	C2—N1—C6—C5	0.2 (5)
O1—C1—C3—C2	4.4 (5)	C2—N1—C6—C6^i^	−179.3 (3)
O2—C1—C3—C4	5.9 (5)	C4—C5—C6—N1	−0.2 (5)
O1—C1—C3—C4	−174.4 (3)	C4—C5—C6—C6^i^	179.2 (3)

Symmetry codes: (i) −*x*, −*y*+1, −*z*+1.

### Hydrogen-bond geometry (Å, °)

**Table:**

*D*—H···*A*	*D*—H	H···*A*	*D*···*A*	*D*—H···*A*
O1—H1···O2^ii^	0.82	1.82	2.625 (3)	168

Symmetry codes: (ii) −*x*+1, −*y*, −*z*+2.
